# Affective States and Cognitive Performance: Results From the Emotions in Daily Life Study

**DOI:** 10.1093/geroni/igaf122.185

**Published:** 2025-12-31

**Authors:** John Castle, Giselle Ferguson, Stacey Scott

**Affiliations:** Stony Brook University, Stony Brook, New York, United States; Union College, Schenectady, New York, United States; Stony Brook University, Stony Brook, New York, United States

## Abstract

Although affective states have been observed to influence cognitive performance in experimental laboratory settings, limited research has investigated this relationship in daily life. In this study, we aimed to understand the relationship between momentary affect (pleasantness, unpleasantness, and intensity) and momentary cognition (processing speed and spatial working memory) using ecological momentary assessment (EMA). For 7 days, 221 participants (Mage=47 years, SD = 15, range=18-82) recruited from an online panel of US adults completed up to 3 EMA per day. In line with laboratory findings, we hypothesized that cognitive performance would be worse at times when individuals experienced more intense, more unpleasant, and less pleasant affective states. We hypothesized age moderation of these effects, such that older age would be associated with weaker momentary affect-cognition slopes. Intraclass correlation coefficients (ICCs) indicated variance both between- and within-persons (ICC processing speed=.66, spatial memory=.58, pleasantness=.32, unpleasantness=.29, intensity=.40). We used separate, parallel multilevel models to assess the affective dimensions’ association with processing speed and spatial working memory, respectively. At times when individuals rated their affect more as more intense than usual, their spatial memory performance was worse (B=.10, p=.03). Unexpectedly, pleasantness and unpleasantness were not associated with cognitive performance either between- or within-persons. Further, we did not find evidence for age as a moderator of these relationships. Thus, in a lifespan sample of adults from across the US, we found that affective intensity, rather than valence, was a key factor associated with cognition in daily life.

